# Testosterone Changes in Couples in Response to a Caregiving Task with an Infant Simulator

**DOI:** 10.1007/s12110-026-09520-5

**Published:** 2026-04-28

**Authors:** Rachel E. Brandon, Annika From, Miranda Reynaga, Helen Devine, Amie M. Gordon, Robin S. Edelstein

**Affiliations:** 1 Department of Psychology, University of Michigan, 530 Church Street, Ann Arbor, MI 48109 USA; 2 School of Social Work, University of Michigan, 1080 S University Ave, Ann Arbor, MI 48109 USA

**Keywords:** Testosterone, Caregiving, Romantic relationships, Infant simulator, Transition to parenthood, Dyadic interaction

## Abstract

In the current study, we assessed the role of caregiving in testosterone reactivity by examining whether and how testosterone changed in romantic partners as they jointly cared for an “infant simulator,” a lifelike doll designed to mimic many of the behaviors of a real infant. Although testosterone often declines as a function of caring for a child, especially when parents feel able to meet the child’s needs, less is known about joint caregiving with romantic partners. Couples without children (*N* = 30; 60 participants) engaged in a joint caregiving task in the laboratory before and after taking care of the infant at home, allowing us to examine how testosterone reactivity changed over time and with caregiving experience. We found that, on average, both female and male participants showed significant post-caregiving declines in testosterone following the second, but not the first, lab session. These findings were not mediated by participants’ feelings about or performance during the at-home caregiving. Results were, however, moderated by participants’ comfort with babies and their expectations about parenting: Participants who were less comfortable with babies or had less positive parenting expectations showed larger differences in reactivity across sessions, suggesting that caregiving experience may have contributed to testosterone declines in the second session. Our findings suggest that those with more caregiving experience or who had more positive expectations about parenting were more easily able to lean into nurturance, perhaps leading to testosterone declines. Taken together, our findings reflect the nuanced, situation-dependent influences on testosterone in dyadic contexts.

**Supplementary Information:**

The online version contains supplementary material available at 10.1007/s12110-026-09520-5.

Testosterone is a steroid hormone that is found in virtually all vertebrates, including birds, reptiles, and mammals (Hirschenhauser & Oliveira, 2006). In humans and other animals, relatively higher versus lower levels of testosterone are thought to be more or less adaptive depending on the social and developmental context (Edelstein, 2022; van Anders et al., 2011; Wingfield et al., 1990): On the one hand, higher testosterone tends to be more beneficial in competitive or aggressive contexts, such as attracting a mate (Archer, 2006; van der Meij et al., 2019); on the other hand, lower testosterone tends to be more beneficial in nurturant or affiliative contexts, such as caregiving (Edelstein et al., 2017; Gray et al., 2002). Differences across contexts are thought to reflect “tradeoffs” in the extent to which testosterone may be more or less adaptive (Edelstein, 2022; van Anders et al., 2011).

Testosterone trade-offs are observed not only at the baseline level—for instance, people with lower endogenous testosterone report more positive feelings about parenting (e.g., Deady et al., 2006), less risk of abusive parenting (Rodriguez et al., 2021), and less aggression toward romantic partners (Kaiser & Powers, 2006)—but also with respect to changes in testosterone or testosterone *reactivity*. Specifically, testosterone tends to decline for both men and women in contexts that elicit nurturance, such as parental care (Edelstein et al., 2019; van Anders et al., 2012; Voorthuis et al., 2019), but tends to increase in contexts that elicit competition or sexual interest, such as sports (Booth et al., 1989) and novel interactions with potential or actual romantic partners (e.g., Chin et al., 2021; Roney & Gettler, 2015; van der Meij et al., 2019).^1^

As highlighted by van Anders et al. (2011), however, caregiving can elicit both defensive and nurturant behavior, depending on the context and emotional state of both the caregiver and care recipient, potentially leading to increases *or* decreases in testosterone. To illustrate this point, van Anders and colleagues (2012) compared men’s testosterone responses to an infant simulator (a lifelike doll that mimics the behavior of a real infant) that had been programmed to be consolable versus inconsolable. In contrast to past findings suggesting uniform testosterone increases in response to baby cries (Fleming et al., 2002; Storey et al., 2000), van Anders and colleagues (2012) found that men’s testosterone increased when they were unable to soothe the infant, but decreased when their efforts to soothe the infant were successful, suggesting that infant cries per se do not elicit testosterone responses. In a similar vein, Froidevaux et al. (2025) examined testosterone changes in expectant couples after they interacted with an inconsolable infant simulator. One couple member was randomly assigned to care for the infant alone for the first five minutes and was then joined by their partner to care for the infant together for the second five minutes. Both mothers and fathers, regardless of who had done the caregiving alone first, showed increases in testosterone after the interaction, again suggesting that parental efficacy may be a critical determinant of testosterone responses.

Taken together, these findings suggest that it may be overly simplistic to think about testosterone as simply increasing or decreasing as a function of caregiving; instead, the infant’s responses, and the parent’s expectations, likely play a role. Specifically, if the context elicits feelings of nurturance (e.g. caring for a crying infant), testosterone may be more likely to decrease; conversely, if the context elicits feelings of protection, defense, or frustration (e.g. being unable to soothe the crying infant), testosterone may be more likely to increase.

To our knowledge, only one study (Froidevaux et al., 2025) has examined the implications of *dyadic* caregiving for testosterone reactivity. All couples in this study engaged with an inconsolable infant, which likely increased participants’ frustration and decreased nurturance. Moreover, this study was focused on links between perceived social support and testosterone reactivity, as opposed to predictors or moderators of testosterone responses per se. In the current study, we sought to better understand when and why testosterone might change among couples, without children, who jointly cared for a consolable infant. We were particularly interested in the extent to which experiences with and feelings about caregiving might influence testosterone changes. Longitudinal research suggests that fathers who are more engaged in childcare show larger pre- to post-fatherhood decreases in testosterone (Gettler et al., 2011); experimental research similarly suggests that fathers who are more comfortable with closeness and intimacy show larger testosterone declines following caregiving experiences (Edelstein et al., 2019). Combined with research on baseline associations between testosterone and interpersonal behavior in both men and women described earlier, we expected that testosterone would be more likely to decline among those with more caregiving interest and experience, including over the course of the study.

The current study reports data from a larger project, in which couples cared for an infant simulator together, allowing us to better isolate the role of nurturance. By recruiting couples without children, we were also able to create a relatively novel situation for both couple members. We assessed the role of caregiving in three ways: First, couples engaged in a semi-structured caregiving task with the infant on two occasions in the lab – once before and once after taking the infant home for the weekend. Presumably, couples would have more experience and comfort with the infant in the second compared to the first session, resulting in fewer feelings of defensiveness or frustration, and potentially leading to larger testosterone decreases in the second session. Second, we assessed potential mediators of any changes in testosterone reactivity from session one to two, including participants’ self-reported feelings about the weekend caregiving and objective performance indicators provided by the infant simulator. Third, we assessed moderators directly related to caregiving: comfort with babies and feelings about parenting. We expected that, insofar as testosterone decreases are driven by nurturance, both female and male participants who were more comfortable with and had more positive expectations about caregiving would show larger testosterone decreases in both sessions.

## Method

### Overview of Study Design

All study procedures were approved by the University of Michigan Institutional Review Board. Data were from a larger study that was designed to simulate the transition to first-time parenthood. Couples (*N* = 31) participated in two laboratory caregiving sessions with an infant simulator—one on a Friday and the second on a Monday (both scheduled at the same time of day). Between the sessions, they cared for the simulator at home. Couples were randomly assigned to either a sleep-deprivation (in which the infant simulator cried regularly throughout the night) or control condition (in which the infant simulator did not cry at all during the night). On Monday, couples returned to the lab for a second caregiving session. During both lab sessions, participants completed self-report measures and provided saliva samples to assess testosterone. Prior to the first lab session, participants completed a series of online background questionnaires (relevant measures are described in more detail below). Additional measures that were collected as part of the study but not considered here are described in the Supplementary Materials. Given the exploratory nature of this work and our reliance on secondary data, we did not conduct a priori power analyses or pre-register our hypotheses or analytic approach.

### Participants and Procedure

Data collection took place from December 2021 to April 2022. To approximate typical demographics of first-time parents, both couple members were required to be between the ages of 20 and 40 and to have lived together for at least one year. Both partners also needed to be available to care for the infant simulator together from Friday afternoon to Monday morning. Eligible couples were those without children (either together or separately; biological or adopted) who intended to become parents in the next five years. Anyone who was pregnant, had diagnosed sleep disorders or medical conditions that could influence hormones (e.g., autoimmune disorders), or smoked was not eligible (Schultheiss & Stanton, 2009). We did not exclude participants taking hormonal contraceptives, given our focus on couples who did not currently have children; 13 female participants reported that they used some form of hormonal contraceptive, 13 reported that they did not, and one did not answer questions about contraceptive use. Given the relatively small size of our sample, we did not examine differences in our findings as a function of contraceptive use. Couples were compensated up to $250 for completing both lab sessions and the at-home portion of the study.

Of the 31 couples who initially agreed to participate, 28 completed the entire study. Of the three couples who did not complete both sessions, one experienced an equipment malfunction with the infant during the at-home portion and declined to complete the rest of the study, one had a scheduling conflict that prevented them from attending the second lab session, and one found the caregiving (especially the sleep disruption) to be too overwhelming, so ended their participation before the second lab session. Data for the three couples with partial completion were included in subsequent analyses as available, resulting in data for 31 couples in the first lab session (30 couples with hormone data), 30 couples for the at-home portion, and 28 couples for the second lab session.

Our analytical sample thus included 30 couples (60 individuals; 29 men, 29 women, 2 agender; 12.9% same-gender couples). To account for what are typically large sex differences in testosterone, we also asked participants to report their sex assigned at birth; we used self-reported sex in subsequent analyses and refer to biological sex rather than gender as relevant. Thirty participants identified as male, and 29 participants identified as female. One participant declined to answer this question; however, for analytical purposes, we inferred their sex assigned at birth as female based on other information reported in the background survey. On average, participants were 27.27 years old (*SD* = 3.56) and self-reported their ethnicity as White (65.0%), Asian (25%), Hispanic or Latinx (5.0%), Black (1.7%), and 3.3% not listed. Participants were highly educated, with over half (55%) holding at least a college degree and over a quarter (28.3%) holding a professional or master’s degree. Most participants were middle class, with 50% reporting an annual household income between $50,000 and $100,000. Relationship length ranged from two to 11 years, with a mean of 5.50 years (*SD* = 2.54). We used participants’ self-reported weight and height to compute Body Mass Index (BMI; *M* = 24.13, *SD* = 5.06).

### Materials and Measures

#### Infant Simulator

The RealCare Baby^®^ is a lifelike infant doll that mimics the behavior of a real infant (Rasmussen et al., 2019); it cries at preset intervals to prompt participants to provide care (i.e., diaper change, feedings, burping, or rocking) using the provided accessories (i.e., a change of clothes, diapers, bottle, blanket, sling, and car seat). As described in more detail below, couples were trained to care for the infant during the first laboratory session. They then cared for the infant over the weekend (Friday night to Monday morning) and engaged in an additional caregiving session during the second lab visit.

#### Background Survey Measures (Completed Before the First Lab Session)

**Comfort with Babies** was assessed with an item created for this study, “How comfortable are you around babies?” Participants responded on a five-point scale, where 1 = “Not comfortable at all” and 5 = “Completely comfortable.”

**Parenting Value** was assessed with items from two subscales of the Life Role Salience Scale (LRSS; Amatea et al., 1986). The 5-item *Parent Role Reward Value* subscale assesses the extent to which parenting is an important means of self-definition and/or personal satisfaction; sample items include “Although parenthood requires many sacrifices, the love and enjoyment of children of one’s own are worth it all.” The 5-item *Parental Role Commitment* subscale assesses the extent to which the person demonstrates a willingness to commit personal resources to assure success in or developing parenting skills; sample items include “I expect to devote a significant amount of my time and energy to the rearing of children of my own.” Items were answered using a five-point scale, where 1 = “Disagree” and 5 = “Agree,” and were averaged to create a total score (α = 0.79).

**Parenting Confidence** was assessed with 14 items adapted from the Karitane Parenting Confidence Scale (Črnčec et al., 2008). Participants were given the prompt, “Please imagine having a child in the future, and indicate how much you agree with the following items” on a four-point scale, where 1 = “Hardly ever” and 4 = “Most of the time.” Items included, “I will know what to do when my baby cries,” and “My partner will be there for me when I need support parenting.” Because the original scale was developed for parents, we modified the items to reference a future rather than current baby. We also excluded one item, “I am confident that my baby is doing well,” because it was not easily translated to a future baby. Per the measure’s manual, items were summed to create a total score (α = 0.82).

#### At-Home Caregiving Measures

##### **Feelings about At-Home Caregiving**

At the beginning of the second lab session, prior to the in-lab caregiving task, participants were asked to report their feelings about the at-home caregiving experience, including how enjoyable, fun, challenging, stressful, tiring, overwhelming, boring, demanding, and effortful they found caring for the infant, as well as how confident and/or uncertain they felt about their performance. Participants provided ratings on a 7-point scale, where 1= “Strongly Disagree” and 7 = “Strongly Agree.” These items were averaged into two subscales: positive feelings towards caregiving (four items; α = 0.81) and negative feelings towards caregiving (seven items; α = 0.92).

##### **Performance Overview**

The infant simulator comes with software that reports how well the couple met the infant’s needs during the at-home care portion of the study. This report includes the number of times the baby was successfully rocked, diapered, burped, or fed, as well as the number of times the baby was not supported in the correct position, held without head support, or shaken. This was a dyad-level score, such that each couple was given one performance score for the weekend caregiving. Scores on this measure ranged from 0 to 100, with higher scores indicating more effective caregiving.

##### **Number of Swipes**

The simulator also provided data on the number of times that each caregiver “swiped” their sensor when caring for the baby, which gives us an index of the frequency of each partner’s contribution to infant care (but not necessarily the effectiveness of that contribution).

#### Caregiving Tasks

Couples engaged twice in a laboratory caregiving task, during which they were left alone to care for the infant together for 10 min; during this time, the infant was programmed to cry every 30 s for 75 s and to require each type of care (i.e., diaper, bottle, burp, soothe). The tasks in the first and second lab sessions were identical, except that couples received training and named their baby prior to the first but not the second session. Lab sessions were videotaped with participants’ consent.

After the first lab session, couples were randomly assigned to one of two conditions: no sleep disruption, in which the infant was programmed to “sleep” (quietly, without crying) throughout the night (9 PM to 8 AM), or moderate sleep disruption, in which the infant was programmed to wake up anywhere from three to six times. Couples were told that, just like real infants, the infant simulators have different sleep schedules, and the infant might cry at night, but they were not made aware of the manipulation or our explicit interest in sleep. Because couples’ experiences during the study were likely influenced by this manipulation, we included experimental condition in all analyses.

#### Salivary Testosterone Collection and Assessment

We collected saliva samples at three points throughout each lab session to measure testosterone, two of which are considered here.^2^ Because hormone levels are most stable in the afternoon to evening hours (Schultheiss & Stanton, 2009), all couples had their lab sessions scheduled between 2:00 PM to 8:00 PM to control for circadian changes in hormones. Both lab sessions were scheduled for the same time of day to minimize variation due to diurnal changes. Participants were instructed to refrain from eating, drinking (except for water), chewing gum, or brushing their teeth for one hour prior to the scheduled session. Participants provided the samples via oral swabs, which they put in their mouths for 60 s and then placed into a plastic tube. This swab method has been validated for testosterone assessment (Bellagambi et al., 2020; Gildner, 2021). Although measuring testosterone this way may artificially increase testosterone levels (though perhaps less so than in other hormones such as DHEA and progesterone; Shirtcliff et al., 2001), this is somewhat less of a concern in our study because we were interested in testosterone changes rather than mean testosterone values and because rank-order stability of testosterone measures obtained with cotton swabs is relatively high (Shirtcliff et al., 2001).

After a 10-minute adaptation period, participants were instructed to provide their first (baseline) saliva sample. Participants then provided their second (post-caregiving) saliva sample 10 min after the end of the 10-minute infant care task. Steroid hormones such as testosterone typically peak approximately 20 min following a stressor or other eliciting event; therefore, this measure was timed to capture physiological reactivity at the beginning of the caregiving task. This time frame is similar to that used successfully with testosterone in prior research (e.g., Chin et al., 2021; Edelstein et al., 2019; Froidevaux et al., 2025; van Anders et al., 2012).

Samples were stored at room temperature until completion of the session and kept at -20 °C until assayed. Samples were analyzed by enzyme immunoassay using a commercial kit from Salimetrics Incorporated. Water-based dilutions of all standards and controls were prepared to determine salivary testosterone concentrations. Samples were assayed in duplicate and the mean levels for each sample were utilized for analysis. Controls were used to assess assay reliability. The inter-assay coefficient of variation (CV) for low and high controls were 16.86% and 9.34%, respectively, for testosterone. The intra-assay CV was 6.87%. Although somewhat high, both inter- and intra-assay CVs are comparable to similar studies conducted in our lab and are in the normal range for published hormone studies (Edelstein et al., 2019; Ketay et al., 2017). Analytic sensitivity (B_0_ – 2 SD) for the two hormonal assays is < 0.007 1 pg/mL. A few participants were missing data at each time point due to low sample volume or collection errors: six in the first session (four pre-task) and three in the second session (all pre-task), so *n*s vary slightly across analyses.

To maximize the use of all available data, hormone values that were larger than three standard deviations above the mean for each sex were winsorized (i.e., replaced with values corresponding to three standard deviations above or below the mean for that particular variable), a commonly used method in the hormone literature (Edelstein et al., 2014; Froidevaux et al., 2025; Ghosh & Vogt, 2012). One (female) participant’s testosterone value from the first lab session was replaced using this approach.^3^ Next, to examine changes in hormones as a function of the caregiving task, we computed percent change scores (i.e., ((Time 2 – Time 1)/Time 1) x 100), a commonly used method of assessing short-term hormone changes (e.g., Edelstein et al., 2019; van Anders et al., 2012). Percent change scores account for baseline differences in hormone levels and are generally preferable to difference scores, which can be difficult to interpret when there are large individual and/or sex differences in baseline hormone levels.

### Analytic Approach

The Statistical Package for the Social Sciences (SPSS, version 30) was used to conduct all analyses. Given the exploratory nature of our analyses, we did not conduct an a-priori power analysis or pre-register our data-analytic plan. We adjusted for nonindependence using a marginal modeling approach in which nonindependence was accounted for within both couples and sessions; we report unstandardized regression coefficients for these analyses. Because our sample included both distinguishable and non-distinguishable dyads (i.e., both mixed- and same-sex couples) and given our relatively small sample size, we treated all dyads as indistinguishable and tested for sex as a moderating variable (Iida et al., 2023). Residual variances and covariances were estimated using a compound symmetry variance-covariance matrix, which provides a single pooled variance estimate for both partners and an estimate of the covariance between partners.

For analyses of testosterone reactivity, we included fixed effects for session, condition, sex, and the session-by-condition interaction. A compound symmetry covariance structure was used to account for within-person correlations across sessions. For all models, session (-1 = session one, 1 = session two), condition (-1 = no sleep deprivation, 1 = sleep deprivation), and actor/partner sex (-1 = female, 1 = male) were contrast coded.

## Results

### Preliminary Analyses

All preliminary analyses were conducted using the marginal modeling approach described above. We report mean pre- and post-task testosterone by condition, sex, and session in Table 1. As expected, in both sessions, male participants had significantly higher testosterone levels than female participants at both pre-task, *t*_*session1*_ (33.09) = 12.79, *p* < .001; *t*_*session2*_ (29.53) = 13.73, *p* < .001, and post-task, *t*_*session1*_ (37.34) = 14.24, *p* < .001; *t*_*session2*_ (33.53) = 12.79, *p* < .001. Male and female participants did not differ in percent change scores at either session, however, *t*_*session1*_ (26.88) = 0.84, *p* = .41; *t*_*session2*_ (38.11) = 0.57, *p* = .57. Pre- and post-task testosterone levels were highly correlated in both sessions for female participants, *r*_*female1*_ = 0.88, *p* < .001, *r*_*female2*_ = 0.84, *p* < .001 and male participants, *r*_*male1*_ = 0.88, *p* < .001, *r*_*male2*_ = 0.91, *p* < .001, indicating significant rank-order stability in hormone levels from before to after the caregiving task. We tested the distribution of all continuous variables, including testosterone, and all were normally distributed (Skewness < |2| and Kurtosis < |7|, which are considered acceptable thresholds for moderate normality in applied research contexts; West et al., 1995).

**Table 1:** Means and standard deviations for testosterone (pg/ml) and percent change in testosterone by session, before and after the caregiving task, sex, and condition

	Lab Session 1						Lab Session 2					
	Pre-Task		Post-Task		% Change		Pre-Task		Post-Task		% Change	
	Control						Control					
	M	SD	M	SD	M	SD	M	SD	M	SD	M	SD
Male	136.08	42.59	138.48	38.79	5.14	23.78	141.54	36.32	120.76	31.77	-13.89	14.56
Female	42.87	14.04	44.60	12.95	1.09	14.02	44.10	13.35	39.82	12.82	-9.57	19.68
	Sleep Disruption						Sleep Disruption					
	M	SD	M	SD	M	SD	M	SD	M	SD	M	SD
Male	150.30	35.88	146.91	36.59	-2.07	13.21	138.39	27.33	132.03	34.47	-5.26	11.21
Female	47.62	19.01	42.80	14.42	-6.79	20.67	50.53	17.13	42.89	14.80	-13.82	16.75
	Total						Total					
Male	144.42	13.72	143.60	36.99	0.60	17.77	139.79	31.03	126.80	33.13	-9.09	13.29
Female	45.51	16.98	43.70	13.50	-3.29	18.15	47.07	15.24	41.24	13.60	-11.53	18.16
Total	96.59	58.18	91.93	57.24	-1.34	17.90	94.30	52.76	84.02	49.93	-10.29	15.76

Sex differences in other key variables were generally small in magnitude and/or not statistically significant: Male and female participants did not differ significantly in their comfort with babies, *t*(36.46) = − 0.86, *p* = .40, parenting confidence, *t*(36.80) = − 0.42, *p* = .68, or negative ratings of at-home care, *t*(28.73) = 0.58, *p* = .57. Female participants reported valuing parenting and enjoying the at-home caregiving more, although neither difference was statistically significant, *t*(30.66) = -1.98, *p* = .06 and *t*(28.56) = -1.85, *p* = .08, respectively.

Correlations among key variables are presented in Table 2. Notably, those with more positive parenting expectations showed larger decreases in testosterone in session 1. Somewhat unexpectedly, negative caregiving evaluations were significantly correlated with testosterone reactivity in lab session 1, indicating that participants who showed increases in testosterone in the first session reported finding the caregiving less stressful; this association was not observed in the second lab session. Interestingly, there were no differences in evaluations by gender, indicating that women and men found the caregiving experience similarly enjoyable and stressful. We also found that people who were more comfortable with babies, those with more parenting confidence, and those who valued parenting more highly found the caregiving experience more enjoyable. Similarly, those who were more comfortable with babies reported more parenting confidence and greater value in parenting; somewhat surprisingly, greater comfort with babies was associated with greater testosterone reactivity in session 2 (but not session 1).

**Table 2:** Descriptive statistics and correlations among key study variables

	1	2	3	4	5	6	7	8	M	SD
1. Testosterone% Change Session 1									-1.34	17.90
2. Testosterone% Change Session 2	0.03								-10.29	15.76
3. Positive Caregiving Evaluation	0.18	0.06							5.08	1.06
4. Negative Caregiving Evaluation	− 0.31*	0.05	− 0.26						3.77	1.44
5. Caregiving Performance	0.28	− 0.10	0.26	− 0.12					80.72	22.12
6. Number of Swipes	− 0.05	− 0.03	0.05	0.10	0.02				25.67	10.74
7. Comfort with Babies	− 0.18	0.44**	0.30*	− 0.13	− 0.03	− 0.10			3.12	1.17
8. Parenting Confidence	− 0.25	0.09	0.33**	− 0.29*	− 0.26	0.01	0.36*		47.79	4.35
9. Parenting Value	− 0.29	0.21	0.47**	− 0.03	0.01	0.17	0.28*	0.33**	3.83	0.73

Note. All correlations were adjusted for nonindependence using the marginal modeling approach described in the statistical analysis section. Caregiving performance is a dyad-level variable, so correlations with this variable reflect couple-level associations. *p *≤* .05, **p < .01

Within-couple correlations in testosterone reactivity were positive but not statistically significant at either session, *r* = .17, *p* = .38, and *r* = .07, *p* = .70, respectively. Testosterone reactivity was also not significantly correlated with BMI, age, time of day, or relationship length, *r*s < |0.17|, *p*s > 0.31, and results were virtually identical when these variables were included; therefore, they were not considered further.

### Changes in Testosterone Following the Caregiving Task

**Fig. 1:**
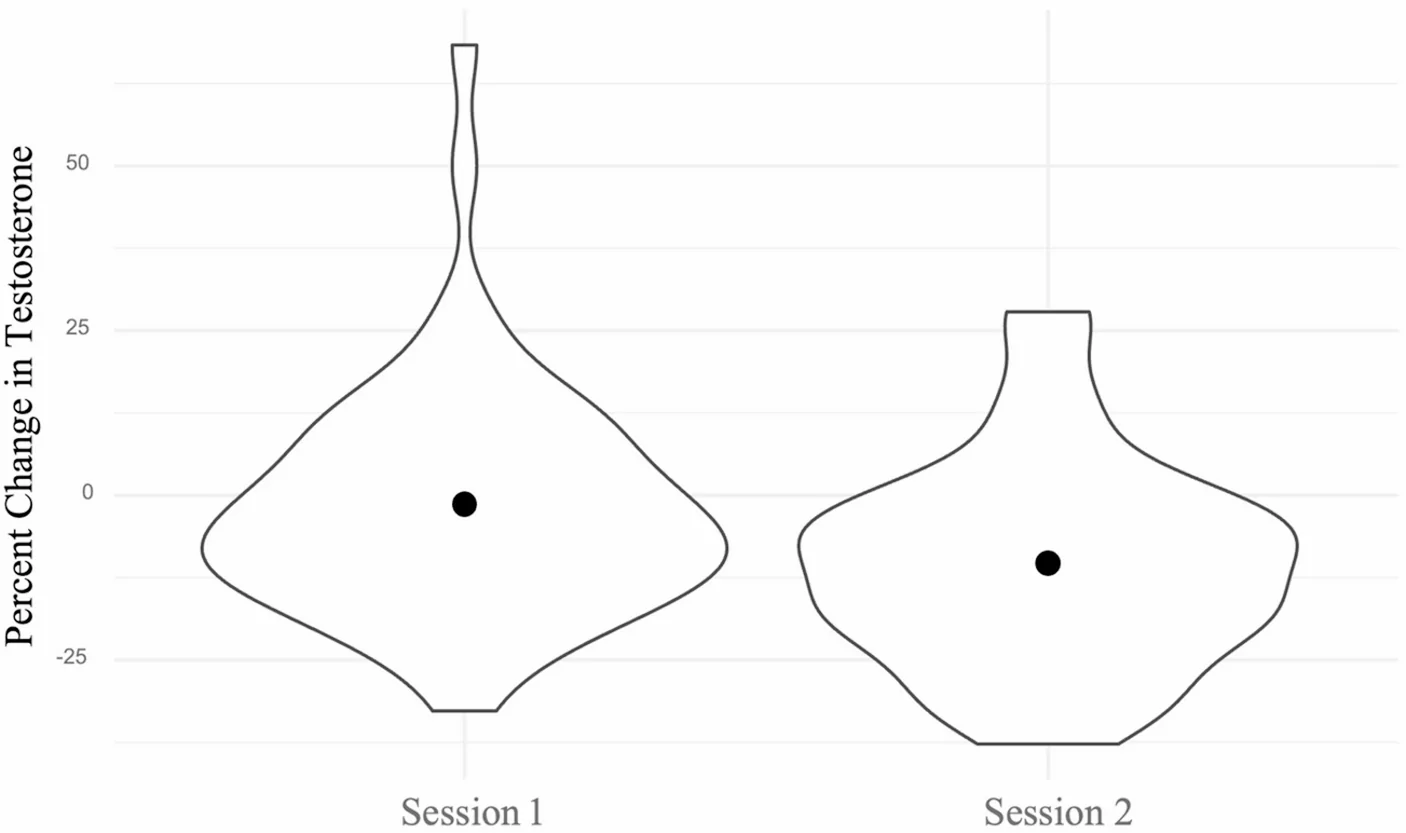
Violin plot of percent change in testosterone by session. *Note*. Center dots represent sample mean

Results from our marginal model (shown in Table 3) revealed a significant effect of session, indicating that testosterone declines were larger in the second compared to the first session. As depicted in Fig. 1, on average, pre-to-post task testosterone decreased in the second session, *t*(26.19) = -5.02, *p* < .001, but average change scores did not differ significantly from zero in the first session, *t*(19.08) = -0.46, *p* = .65. Of note, there was variability in testosterone reactivity in both sessions, suggesting that some participants may have experienced increases while others experienced decreases. The effects of condition, actor/partner sex, and the session-by-experimental condition were not statistically significant.^4^

**Table 3:** Multilevel models predicting percent change in testosterone

	Estimate (SE)	df	t	*p*	CI
Intercept	-5.50 (1.65)	43.90	-3.33	0.002	-8.83, -2.17
Session	-4.84 (1.65)	43.89	-2.93	0.005	-8.17, -1.51
Actor Sex	-1.17 (2.34)	55.45	-0.50	0.62	-5.85, 3.51
Partner Sex	0.71 (2.33)	55.32	0.30	0.76	-3.97, 5.38
Condition	-1.24 (1.69)	44.04	-0.73	0.47	-4.63, 2.16
Session * Condition	2.41 (1.65)	43.88	1.46	0.15	− 0.92, 5.74

Note. Estimates are unstandardized regression coefficients. Sex: 1 = female, -1 = male. Condition: -1 = control, 1 = sleep disruption

### Testing for Mediators of Differences between Sessions

We next examined whether differences in testosterone reactivity across sessions might be explained by differences in participants’ evaluations of their experiences at home with the infant simulator, including positive and negative feelings towards the caregiving experience, as well as the caretaking performance score from the simulator (an indicator of how reliably parents met the baby’s needs).

To test mediation, we first computed a difference score (session 1% change in testosterone – session 2% change in testosterone), such that higher scores reflected a larger difference (decrease) in testosterone reactivity from the first to the second session. We then re-ran our original model, excluding session as a fixed effect, predicting this difference score from the three (centered) potential mediators—positive and negative caregiving evaluations, performance overview, and number of swipes—actor and partner sex, and experimental condition. None of the potential mediators were significant in this model, *b*_*PositiveEval*_ = -2.00, *t*(19.42) = − 0.53, *p* = .60; *b*_*NegativeEval*_ = -3.12, *t*(19.76) = -1.09, *p* = .29; *b*_*Performance*_ = 0.294, *t*(10.81) = 1.34, *p* = .21; *b*_*Swipes*_ = 0.13, *t*(18.64) = 0.32, *p* = .75, including when each mediator was tested in a separate model, all *p*s > 0.23. Therefore, participants’ experience with caregiving over the weekend did not appear to explain differences in testosterone reactivity between sessions.

#### Moderators: Comfort with Babies and Expectations about Parenting

Next, we examined whether participants who were more comfortable with babies and/or had more positive expectations about parenting might be more likely to show the post-caregiving declines in testosterone observed in prior work with parents and children. To test for moderation, we included the interactions between session and (1) comfort with babies, (2) parenting value, and (3) parenting confidence, separately, in the marginal models described earlier. All variables were centered prior to analysis. Simple slopes for significant interactions were probed following Preacher et al. (2003).

**Fig. 2:**
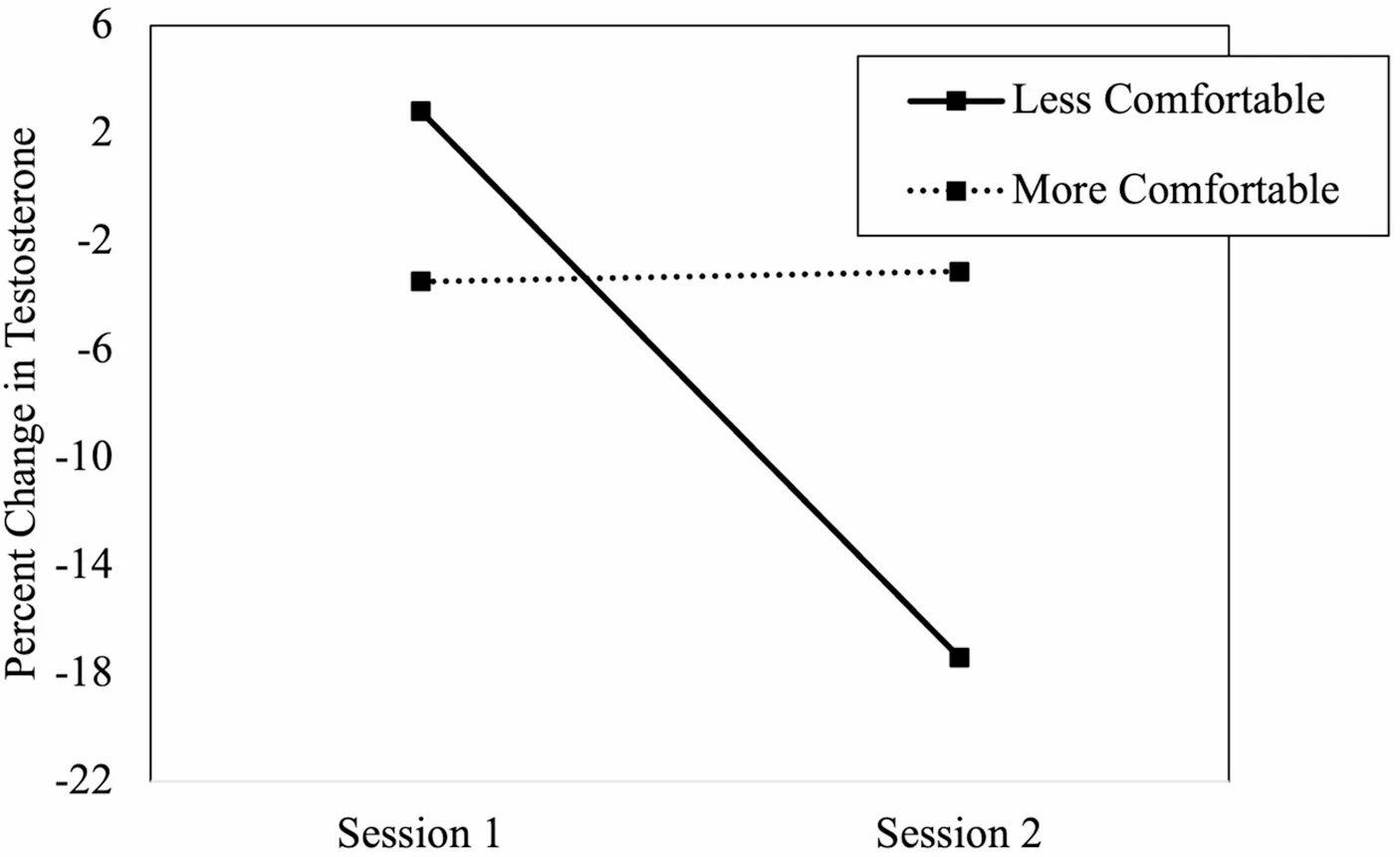
Percent change in testosterone by session for participants reporting low and high comfort with babies. *Note*. Simple slopes are plotted at one standard deviation above and below the mean of comfort with babies

First, in addition to the significant effect of session, reported above, *b* = -4.86, *t*(40.48) = -2.95, *p* = .005, we found a significant interaction between comfort with babies and session, *b* = 4.37, *t*(98.67) = 3.18, *p* = .002. The main effect of comfort with babies was positive but not statistically significant, *b* = 1.81, *t*(98.71) = 1.30, *p* = .20. As depicted in Fig. 2, analyses of simple slopes revealed that participants who were more comfortable with babies showed a similar pattern of testosterone change across sessions, *b* = 0.20, *z* = 0.09, *p* = .93; those who were less comfortable with babies showed a significantly larger decline in testosterone in session 2 compared to session 1, *b* = -9.95, *z* = -4.31, *p* < .001. Put another way, participants who were more comfortable with babies generally showed a decline in testosterone in both sessions, whereas those who were less comfortable showed a decline only in the second session, suggesting that the experience of caring for the infant at home may have had a greater impact on the second laboratory caregiving experience for those less experienced with infants. Further, the difference between those who were more and less comfortable with infants was statistically significant in session 2, *b* = 6.18, *z* = 3.09, *p* = .002 (also reflected in the zero-order correlations), but not in session 1, *b* = -2.56, *z* = -1.35, *p* = .18. No other effects were significant in this analysis, all *p*s > 0.22.

**Fig. 3:**
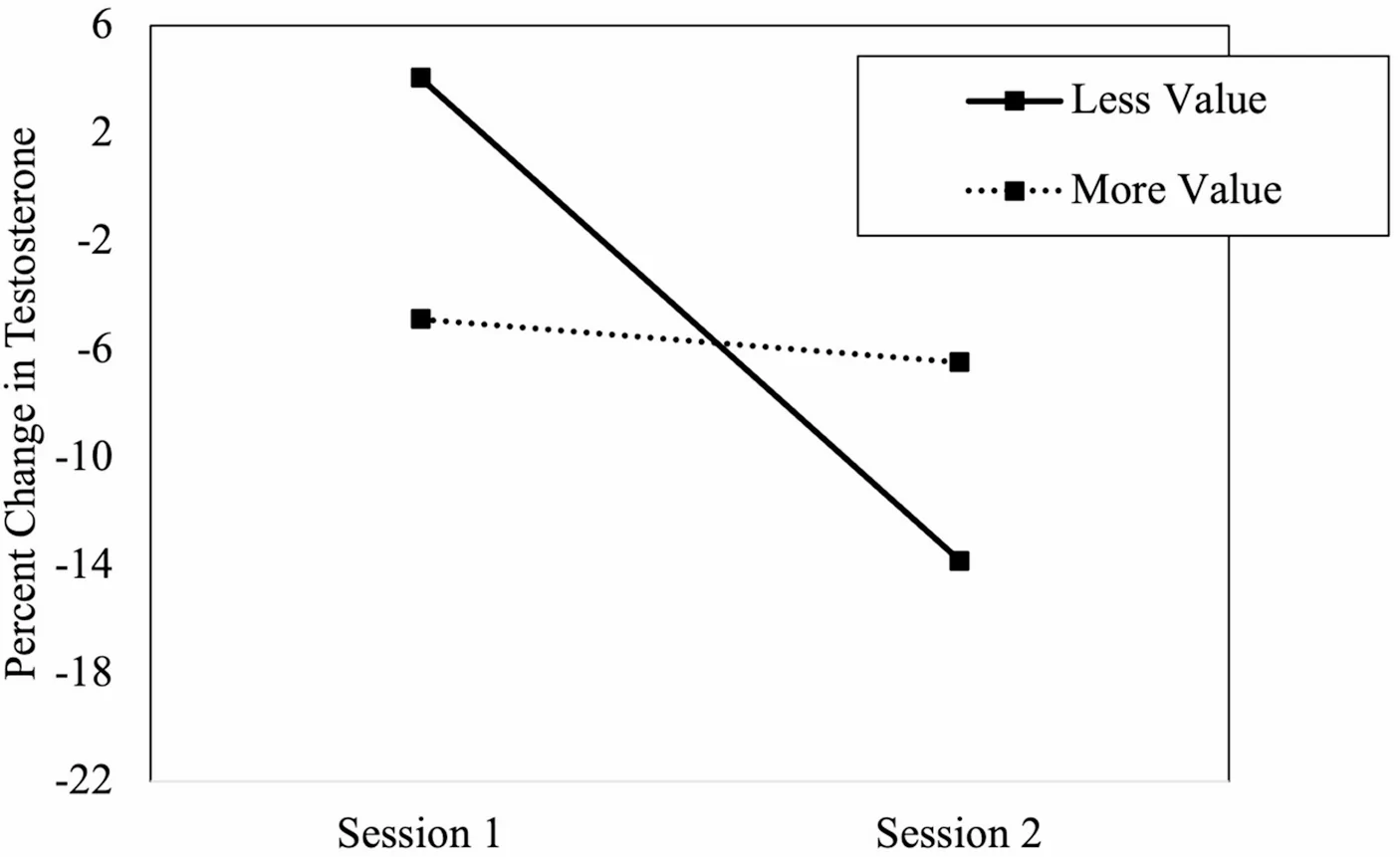
Percent change in testosterone by session for participants placing low versus high value on parenting. *Note*. Simple slopes are plotted at one standard deviation above and below the mean of parenting value

Next, in addition to the significant effect of session, *b* = -4.87, *t*(42.79) = -2.88, *p* = .006, we found a significant interaction between session and parenting value, *b* = 5.66, *t*(63.93) = 2.43, *p* = .02. The main effect of parenting value was not statistically significant, *b* = -0.55, *t*(61.92) = − 0.23, *p* = .82. As depicted in Fig. 3, participants who valued parenting more showed similar patterns of testosterone change across sessions, *b* = -0.79, *z* = -0.33, *p* = .74; those who valued parenting less showed a significantly greater decline in testosterone reactivity in session 2 compared to session 1, *b* = -8.94, *z* = -3.75, *p* < .001. Thus, similar to our findings for comfort with babies, participants who placed more value on parenting showed similar declines in testosterone in both sessions, whereas those who placed less value on parenting showed a decline only in the second session. No other effects were significant in this analysis, all *p*s > 0.19.

Finally, in the model including parenting confidence, we again found a significant effect of session, *b* = -4.87, *t*(43.28) = -2.98, *p* = .005. The interaction between session and parenting confidence was in the same direction as those reported above but was not statistically significant, *b* = 0.68, *t*(99.00) = 1.84, *p* = .07. The main effect of parenting confidence was not statistically significant, *b* = -0.35, *t*(98.62) = − 0.92, *p* = .36, and no other effects were significant in this analysis, all *p*s > 0.14.^5^,^6^

## Discussion

In the current study, we assessed the role of caregiving experience in testosterone reactivity by examining whether and how testosterone changed, in both members of a couple, while they jointly cared for an infant simulator. Previous research generally suggests that nurturant interactions with children lead to testosterone declines in both men and women (e.g., Edelstein et al., 2019), particularly when people feel that they can meet the child’s needs (van Anders et al., 2012). Although much less is known about *dyadic* caregiving, one study found that testosterone *increased* when couples jointly cared for an inconsolable infant (Froidevaux et al., 2025).

To better understand the mechanisms that might lead to testosterone decreases in some contexts and increases in others, we assessed testosterone reactivity across two laboratory caregiving sessions, separated by a weekend of at-home caregiving, which allowed us to examine the role of caregiving experience and familiarity with the infant simulator. By focusing on a joint caregiving task, with an infant, we aimed to separate the role of nurturance specifically from other romantic relationship processes that might be involved in interactions between couple members, such as sexual attraction (e.g., Chin et al., 2021). We assessed potential mediators of any changes in testosterone reactivity from session one to two, including participants’ self-reported feelings about the at-home caregiving and objective performance indicators provided by the infant simulator (i.e., performance overview and number of swipes). We also measured three moderators directly related to caregiving: comfort with babies, value placed on parenting, and confidence about parenting.

We found that, on average, both female and male participants showed significant post-caregiving declines in testosterone following the second, but not the first, lab session. The testosterone declines in the second session are consistent with previous research on parent-child interactions, suggesting that these interactions may have been experienced as more nurturant and/or less challenging than those in the first laboratory session. Of note, our findings did not differ by sleep-deprivation condition, suggesting that sleep loss was unlikely to have contributed to differences across sessions, although our sample may not have been sufficiently powered to detect small differences. Perhaps because couples had experience with the infant simulator between the two sessions, the second caregiving task may have seemed less confusing, stressful, or novel. Such findings would be consistent with previous literature suggesting that testosterone reactivity depends on the caregiver’s sense of efficacy (van Anders et al., 2012). In a similar vein, research on testosterone reactivity in romantic partners suggests that novel tasks (e.g., disclosing previously unknown information to a romantic partner) may lead to increases in testosterone due, in part, to increases in emotional intimacy and sexual attraction (Chin et al., 2021). In other words, insofar as participants found the first caregiving task more novel or exciting than the second, they may have been less likely to show testosterone decreases in the first session.

To test the possibility that participants’ feelings about the infant simulator or caregiving performance contributed to differences in testosterone reactivity across sessions, we examined several potential mediators from the at-home caregiving portion of the study: participants’ positive and negative feelings towards the caregiving experience and the infant-provided performance score of caregiving effectiveness. Somewhat counterintuitively, participants who showed testosterone increases found the at-home caregiving less stressful and performed better at it; perhaps, in this context, small increases in testosterone reflected a challenge or protective response that was helpful in terms of at-home caregiving. None of the variables we tested mediated the difference between sessions, however, leaving the mechanisms underlying change across sessions unclear. Future research might further investigate these differences by directly assessing or experimentally manipulating task novelty or caregiving efficacy.

To further examine the role of caregiving experience, we tested three moderators of testosterone reactivity and differences in reactivity across sessions: comfort with babies, value placed on parenting, and parenting confidence (measured prior to the caregiving sessions in the lab). Comfort with babies and parenting value significantly moderated the effect of session on testosterone changes: People who reported being more comfortable with babies showed similar declines in testosterone in both sessions, whereas those who reported less comfort with babies showed declines in testosterone only in session 2. Similarly, people who placed more value on parenting showed similar declines in testosterone across sessions, whereas people who had less positive parenting expectations showed significant declines in testosterone only in session 2. Parenting confidence showed a similar pattern, but the interaction effect was not statistically significant. Perhaps those with more caregiving experience or more positive expectations about parenting were more easily able to lean into nurturance, which led to the declines in testosterone that are typically observed among more experienced caregivers. That people who rated themselves as less comfortable with babies and those who had less positive parenting expectations showed testosterone declines similar to their more comfortable counterparts in session 2 suggests that participants acclimated to baby care relatively quickly. Importantly, however, because we did not measure or manipulate perceptions of efficacy (e.g., by manipulating soothability, van Anders et al., 2012), our findings cannot speak to the role of efficacy per se in testosterone changes.

Of course, our results cannot be generalized to hormone changes among parents of actual infants, as our participants were interacting with an infant simulator. Further, although our sample size is comparable to other studies employing infant simulators (e.g., van Anders et al., 2012), our sample was nevertheless relatively small and homogenous, making it difficult to generalize our findings to broader populations. In particular, although our sample included both mixed- and same-sex couples, the majority of couples were mixed-sex, limiting our ability to assess differences between couple types. Replication in a larger sample would help clarify our findings and determine any differences by couple type. Despite our relatively small sample size, we believe the intensive data collection, range of methods (e.g., hormones, infant-simulator-recorded performance scores, self-reports), as well as our within-subject design (which increases statistical power), lend credibility to our findings. Future studies would also be strengthened by pre-registering hypotheses and conducting a-priori power analyses to ensure sufficient statistical power. Finally, while we hypothesize that novelty may have contributed to the difference between sessions, we did not measure novelty per se, so we could not test this possibility directly. Future researchers might consider a similar study design, with the addition of a control condition in which couples do not spend the weekend with the infant, to explicitly test the effects of habituation and practice on testosterone reactivity.

Despite these limitations, our findings contribute to knowledge about changes in testosterone in response to caregiving in a dyadic context. Consistent with research and theory suggesting that testosterone reactivity may depend on the extent to which an interaction is experienced as nurturant versus challenging, we found that caregiving experience – both at the trait- and situational-level, led to testosterone declines following caregiving events. Taken together, our findings highlight the complex, nuanced, and situation-dependent role of testosterone in dyadic contexts.

## Electronic Supplementary Material

Below is the link to the electronic supplementary material.

Supplementary Material 1

## Data Availability

Key variables used in the current study are available upon request. Syntax for our main analyses can be found at [https://osf.io/dzb7m/files/osfstorage](https:/osf.io/dzb7m/files/osfstorage) .

## Footnotes

1 Note that we use “women” and “men” when reporting findings from studies that use this language.

2 The third saliva sample was obtained after a subsequent task, which involved a problem-solving conversation, with the goal of capturing changes as a function of that particular conversation. Because responses at the third time point were likely influenced by the problem-solving conversation (as intended), we did not include that sample in our analyses.

3 Results were virtually identical when we used the unwinsorized values to compute percent change scores.

4 We also initially included the two-way interactions between actor/partner sex and session; however, these effects were relatively small in magnitude and none of these interactions were statistically significant, *b*_*ActorSex*Session*_ = − 0.10, *t*(54.35) = − 0.04, *p* = .97; *b*_*PartnerSex*Session*_ = − 0.92, *t*(54.23) = − 0.39, *p* = .70, respectively, so they were removed in final analyses.

5 As shown in Table 2, the three potential moderators were moderately positively correlated. A similar interaction with session emerged when these variables were averaged to create one composite moderator, *b* = 2.31, *t* (96.51) = 2.53, *p* = .01; participants who scored higher on the composite showed a smaller difference in testosterone change across sessions, *b* = − 0.84, *z* = − 0.39, *p* = .13, compared to those who scored lower, *b* = -8.87, *z* = -3.70, *p* < .001.

6 Results were similar when we ran the model with raw testosterone values (rather than percent change); however, the moderation results did not reach statistical significance.

## References

[CR1] Amatea, E. S., Cross, E. G., Clark, J. E., & Bobby, C. L. (1986). Assessing the work and family role expectations of career-oriented men and women: The life role salience scales. *Journal of Marriage and the Family*, 831–838.

[CR2] Archer, J. (2006). Testosterone and human aggression: An evaluation of the challenge hypothesis. *Neuroscience & Biobehavioral Reviews*, *30*(3), 319–345. 16483890 10.1016/j.neubiorev.2004.12.007

[CR3] Bellagambi, F. G., Lomonaco, T., Salvo, P., Vivaldi, F., Hangouët, M., Ghimenti, S., Biagini, D., Di Francesco, F., Fuoco, R., & Errachid, A. (2020). Saliva sampling: Methods and devices. An overview. *TrAC Trends in Analytical Chemistry*, *124*, 115781.

[CR4] Booth, A., Shelley, G., Mazur, A., Tharp, G., & Kittok, R. (1989). Testosterone, and winning and losing in human competition. *Hormones and Behavior*, *23*, 556–571. 2606468 10.1016/0018-506x(89)90042-1

[CR5] Chin, K., Reese, Z., Ascigil, E., Sim, L., & Edelstein, R. S. (2021). Closeness-inducing discussions with a romantic partner increase cortisol and testosterone. *Psychoneuroendocrinology*, *132*, 105357. 34303223 10.1016/j.psyneuen.2021.105357

[CR7] Deady, D., Smith, M., Sharp, M., & Al-Dujaili, E. (2006). Maternal personality and reproductive ambition in women is associated with salivary testosterone levels. *Biological Psychology*, *71*, 29–32. 16360878 10.1016/j.biopsycho.2005.01.009

[CR8] Edelstein, R. S. (2022). Testosterone tradeoffs in close relationships. *Advances in Experimental Social Psychology*, *65*, 235–280.

[CR9] Edelstein, R. S., Chin, K., Saini, E. K., Kuo, P. X., Schultheiss, O. C., & Volling, B. L. (2019). Adult attachment and testosterone reactivity: Fathers’ avoidance predicts changes in testosterone during the Strange Situation Procedure. *Hormones and Behavior*, *112*, 10–19. 30879994 10.1016/j.yhbeh.2019.01.009 PMC7328342

[CR10] Edelstein, R. S., Chopik, W. J., Saxbe, D. E., Wardecker, B. M., Moors, A. C., & LaBelle, O. P. (2017). Prospective and dyadic associations between expectant parents’ prenatal hormone changes and postpartum parenting outcomes. *Developmental Psychobiology*, *59*, 77–90. 27604815 10.1002/dev.21469 PMC5313241

[CR11] Edelstein, R. S., van Anders, S. M., Chopik, W. J., Goldey, K. L., & Wardecker, B. M. (2014). Dyadic associations between testosterone and relationship quality in couples. *Hormones and Behavior*, *65*, 401–407. 24650800 10.1016/j.yhbeh.2014.03.003

[CR12] Fleming, A. S., Corter, C., Stallings, J., & Steiner, M. (2002). Testosterone and prolactin are associated with emotional responses to infant cries in new fathers. *Hormones and Behavior*, *42*(4), 399–413. 12488107 10.1006/hbeh.2002.1840

[CR13] Froidevaux, N. M., Lai, J., Simon, S. G., Benjamin, L., Somers, J. A., Granger, D. A., … & Borelli, J. L. (2025). The dyadic association of testosterone with perceived social support in couples across the transition to parenthood. *Developmental Psychobiology*, *67*(5), e70078. 10.1002/dev.70078 PMC12464473 40999715

[CR34] Gettler, L. T., McDade, T. W., Feranil, A. B., & Kuzawa, C. W. (2011). Longitudinal evidence that fatherhood decreases testosterone in human males. *Proceedings of the National Academy of Sciences, 108*, 16194–16199. 10.1073/pnas.1105403108 PMC3182719 21911391

[CR15] Ghosh, D., & Vogt, A. (2012, July). Outliers: An evaluation of methodologies. In Joint statistical meetings (Vol. 12, No. 1, pp. 3455–3460).

[CR14] Gildner, T. E. (2021). Reproductive hormone measurement from minimally invasive sample types: Methodological considerations and anthropological importance. *American Journal of Human Biology*, 33(1), e23535. 33174269 10.1002/ajhb.23535

[CR16] Gray, P. B., Kahlenberg, S. M., Barrett, E. S., Lipson, S. F., & Ellison, P. T. (2002). Marriage and fatherhood are associated with lower testosterone in males. *Evolution and Human Behavior*, *23*, 193–201.

[CR17] Hirschenhauser, K., & Oliveira, R. F. (2006). Social modulation of androgens in male vertebrates: Meta-analyses of the challenge hypothesis. *Animal Behaviour*, *71*, 265–277.

[CR18] Iida, M., Savord, A., & Ledermann, T. (2023). Dyadic longitudinal models: A critical review. *Personal Relationships*, *30*, 356–378.

[CR19] Kaiser, H., & Powers, S. (2006). Testosterone and conflict tactics within late-adolescent couples: a dyadic predictive model. *Journal of Social and Personal Relationships*, *23*, 231–248.

[CR20] Ketay, S., Welker, K. M., & Slatcher, R. B. (2017). The roles of testosterone and cortisol in friendship formation. *Psychoneuroendocrinology*, *76*, 88–96. 27898358 10.1016/j.psyneuen.2016.11.022

[CR21] Preacher, K. J., Curran, P. J., & Bauer, D. J. (2003). *Simple intercepts, simple slopes, and regions of significance in MLR 2-way interactions*. https://www.quantpsy.org/interact/hlm2.htm

[CR22] Rasmussen, H. F., Corner, G. W., & Margolin, G. (2019). Young adult couples’ behavioral and physiological responses to the infant simulator: A preliminary illustration of coparenting. *Infant Behavior and Development*, *56*, 101255. 29728255 10.1016/j.infbeh.2018.04.004

[CR6] Črnčec, R., Barnett, B., & Matthey, S. (2008). Karitane Parenting Confidence Scale. *Sydney, Australia: Sydney South West Area Health Service*.

[CR23] Rodriguez, C. M., Granger, D. A., & Leerkes, E. M. (2021). Testosterone associations with parents’ child abuse risk and at-risk parenting: A multimethod longitudinal examination. *Child Maltreatment*, *26*, 50–62. 32500732 10.1177/1077559520930819 PMC7718373

[CR24] Roney, J. R., & Gettler, L. T. (2015). The role of testosterone in human romantic relationships. *Current Opinion in Psychology*, *1*, 81–86.

[CR25] Schultheiss, O. C., & Stanton, S. J. (2009). Assessment of salivary hormones. In E. Harmon-Jones & J. S. Beer (Eds.), *Methods in Social Neuroscience* (pp. 17–44). Guilford Press.

[CR26] Shirtcliff, E. A., Granger, D. A., Schwartz, E., & Curran, M. J. (2001). Use of salivary biomarkers in biobehavioral research: Cotton-based sample collection methods can interfere with salivary immunoassay results. *Psychoneuroendocrinology*, *26*, 165–173. 11087962 10.1016/s0306-4530(00)00042-1

[CR27] Storey, A. E., Walsh, C. J., Quinton, R. L., & Wynne-Edwards, K. E. (2000). Hormonal correlates of paternal responsiveness in new and expectant fathers. *Evolution and Human Behavior*, *21*, 79–95. 10.1016/s1090-5138(99)00042-2 10785345

[CR28] van Anders, S. M., Goldey, K. L., & Kuo, P. X. (2011). The Steroid/Peptide Theory of Social Bonds: Integrating testosterone and peptide responses for classifying social behavioral contexts. *Psychoneuroendocrinology*, *36*, 1265–1275. 21724336 10.1016/j.psyneuen.2011.06.001

[CR29] van Anders, S. M., Tolman, R. M., & Volling, B. L. (2012). Baby cries and nurturance affect testosterone in men. *Hormones and Behavior*, *61*, 31–36. 22001872 10.1016/j.yhbeh.2011.09.012

[CR30] van der Meij, L., Demetriou, A., Tulin, M., Méndez, I., Dekker, P., & Pronk, T. (2019). Hormones in speed-dating: The role of testosterone and cortisol in attraction. *Hormones and Behavior*, *116*, 104555. 31348926 10.1016/j.yhbeh.2019.07.003

[CR31] Voorthuis, A., Bakermans-Kranenburg, M. J., & Van IJzendoorn, M. H. (2019). Testosterone reactivity to infant crying and caregiving in women: the role of oral contraceptives and basal cortisol. *Infant Behavior and Development*, *56*, 101191. 28830625 10.1016/j.infbeh.2017.08.002

[CR32] West, S. G., Finch, J. F., & Curran, P. J. (1995). Structural equation models with nonnormal variables: Problems and remedies. In R. H. Hoyle (Ed.), *Structural equation modeling: concepts, issues, and applications* (pp. 56–75). Sage.

[CR33] Wingfield, J. C., Hegner, R. E., Dufty, A. M., & Ball, G. F. (1990). The challenge hypothesis: Theoretical implications for patterns of testosterone secretion, mating systems, and breeding strategies. *The American Naturalist*, *136*, 829–846.

