# Supplementary material for: Testosterone Changes in Couples in Response to a Caregiving Task with an Infant Simulator
Source: Hum Nat. 2026 Apr 28;37(2):172–91. doi: 10.1007/s12110-026-09520-5 (PMC13506511; doi:10.1007/s12110-026-09520-5)
Supplement: Supplementary file 1 [file 12110_2026_9520_MOESM1_ESM.docx]

**Supplementary Materials**

**Table S1**

*Multilevel Models Predicting Percent Change in Testosterone – Female Participants Only*

|  | Estimate (*SE*) | *df* | *t* | *p* | *CI* |
| --- | --- | --- | --- | --- | --- |
| Intercept | -9.84 (3.21) | 44.44 | -3.07 | .004 | -16.30, -3.38 |
| Partner Sex | -3.71 (3.21) | 44.44 | -1.16 | .25 | -10.18, 2.75 |
| Session | -4.41 (2.30) | 92.14 | -1.92 | .06 | -8.97, .15 |
| Condition | -3.07 (2.30) | 92.02 | -1.34 | .19 | -7.63, 1.49 |
| Session * Condition | 1.00 (2.30) | 92.20 | .43 | .67 | -3.57, 5.56 |

*Note.* Estimates are unstandardized regression coefficients. Partner Sex: 1 = female, -1 = male. Condition: -1 = control, 1 = sleep disruption

**Table S2**

*Multilevel Models Predicting Percent Change in Testosterone – Male Participants Only*

|  | Estimate (*SE*) | *df* | *t* | *p* | *CI* |
| --- | --- | --- | --- | --- | --- |
| Intercept | -9.12 (3.45) | 51.22 | -2.64 | .01 | -16.04, -2.19 |
| Partner Sex | 6.81 (3.41) | 50.08 | 2.00 | .05 | -.04, 13.66 |
| Session | -5.45 (2.31) | 94.36 | -2.36 | .02 | -10.04, -.86 |
| Condition | 2.06 (2.46) | 96.82 | .84 | .40 | -2.82, 6.95 |
| Session * Condition | 4.08 (2.31) | 94.36 | 1.77 | .08 | -.51, 8.67 |

*Note.* Estimates are unstandardized regression coefficients. Partner Sex: 1 = female, -1 = male. Condition: -1 = control, 1 = sleep disruption

**Additional Information**

As part of the larger study, couples also completed several additional measures that are not considered further here, including twice-daily, at-home surveys about their experiences with the infant, daily at-home saliva samples to assess cortisol levels, and electrophysiological data throughout each lab session. Following the caregiving task, couples also completed conflict and gratitude conversations in each lab session.
